# The Future of Technology in Positive Psychology: Methodological Advances in the Science of Well-Being

**DOI:** 10.3389/fpsyg.2018.00962

**Published:** 2018-06-18

**Authors:** David B. Yaden, Johannes C. Eichstaedt, John D. Medaglia

**Affiliations:** ^1^Department of Psychology, University of Pennsylvania, Philadelphia, PA, United States; ^2^Department of Neurology, University of Pennsylvania, Philadelphia, PA, United States; ^3^Department of Psychology, Drexel University, Philadelphia, PA, United States

**Keywords:** positive psychology, psychopharmacology, non-invasive brain stimulation, computational linguistics, virtual reality, technology

## Abstract

Advances in biotechnology and information technology are poised to transform well-being research. This article reviews the technologies that we predict will have the most impact on both measurement and intervention in the field of positive psychology over the next decade. These technologies include: psychopharmacology, non-invasive brain stimulation, virtual reality environments, and big-data methods for large-scale multivariate analysis. Some particularly relevant potential costs and benefits to individual and collective well-being are considered for each technology as well as ethical considerations. As these technologies may substantially enhance the capacity of psychologists to intervene on and measure well-being, now is the time to discuss the potential promise and pitfalls of these technologies.

## Introduction

In *Homo Deus*, historian Harari (2016) extrapolates from current trends to predict how technology might influence humanity’s future. As society continues to succeed in reducing disease, poverty, and violence (e.g., Pinker, 2011, 2018), Harari argues that more resources will likely be devoted to extending the human life span and fostering well-being. In terms of well-being, Harari points specifically to the increasing capacity of technologies to modulate mental states and for algorithms to help guide decision making in ways highly relevant to well-being. According to Harari, these advances in biological and information technology may exert such a substantial influence on the well-being of those who use them that these individuals could have a fundamentally different experience of life. In other words, enhanced individuals may differ from the non-enhanced not only in degree – but also in kind.

We are still far from any such fundamental alterations to human experience, but there are several technologies poised to profoundly influence the scientific study of well-being, even within the next decade. While Harari provides fascinating far-future speculations about psychopharmacology, non-invasive brain stimulation, virtual reality environments, and big-data methods, there is a need to review and discuss *current and emerging* manifestations of these technologies. In this perspective article, we predict that the technologies we review will become commonplace in psychological research on well-being in this decade, yet many well-being researchers are relatively unaware of them or ill-informed about their potential. In this review, we use Harari’s work as an inspiration to describe some of these technologies and call for further ethical and safety discourse due to the increased capacity for intervention and measurement that these technologies make possible.

The most well-known umbrella term for the scientific study of well-being is Positive Psychology (Satterfield, 2000; Seligman, 2018). While Seligman’s (1998) address to the APA on balancing an overemphasis on psychopathology to include more research on well-being marks an important moment for the field of Positive Psychology, quantitative well-being research (e.g., Diener, 1984) and positive psychology theory (e.g., Maslow, 1964) stretch back decades earlier. In recent years, some progress has been made toward understanding the major contributors to, and other outcomes associated with, well-being. Some reliable factors associated with well-being include; income, physical health, marriage, optimism, and social support (e.g., Diener et al., 2002). Technological advances will likely support, advance, and in some cases provide entirely new tools for well-being measurement and intervention.

In terms of current measurement tools, well-being research typically uses self-report surveys distributed through digital platforms. While this was once a paper-and-pencil process, survey data are now typically gathered through digital surveys hosted on platforms like Qualtrics and Amazon’s Mechanical Turk or posted on websites (Buhrmester et al., 2011). Unlike in previous decades, most psychometric scales distributed in contemporary research have been tested for their factor structure, validity, and reliability – and are used only after having demonstrated adequacy across several metrics (Furr, 2011). The most well known scales in positive psychology can be generally divided into affective measures and satisfaction measures. An example of an affective measure is *Positive and Negative Affect Scale* (*PANAS*; Watson et al., 1988) which asks participants to indicate the emotions that they have recently experienced (Watson et al., 1988) and an example of a satisfaction measure is *Satisfaction With Life Scale* (SWLS; Diener et al., 1985) which asks participants to indicate their overall assessment of their life. While other measures of well-being exist, such as measuring facial expressions like Duchenne smiles (Ekman et al., 2013) and some physiological and neurological markers (Davidson and Irwin, 1999), self-report scales distrusted through online survey platforms is the most common measurement strategy among contemporary psychological researchers.

In terms of current well-being interventions, sometimes called “positive interventions” (Rashid, 2009), most consist of psychosocial activities. Like cognitive behavioral therapy (CBT; Beck, 1979), many of these interventions presume that a change in attention, engagement, and beliefs can foster both a change in behavior and emotional experience. One example of a positive intervention is “Three Good Things,” in which one keeps track of three good things that happened to them and why (Seligman et al., 2005). Structured well-being-oriented curricula have been developed, including the Penn Resiliency Program, which teaches children psychological skills that have been demonstrated to reduce the incidence of mental health problems (Gillham et al., 2007), Comprehensive Soldier Fitness (Cornum et al., 2011), which is aimed at increasing the resilience of the U.S. armed forces, as well as various positive education initiatives, which aim to increase psychosocial skills, mindfulness, and aspects of character development, have shown success in improving academic performance and well-being in some countries (e.g., Adler, 2017). Positive interventions have generally been demonstrated to be reliably effective yet small in effect size (Seligman et al., 2005; Bolier et al., 2013). Notably, our interest in positive interventions in this article is descriptive rather than prescriptive; we see positive interventions primarily as tools with which to scientifically study well-being.

Measurement and intervention paradigms in well-being research are on the verge of shifting, however, and the current measurement and intervention tools described in the previous two paragraphs will likely look quite different a decade from the time of this writing. These changes are largely due to several technologies that will likely exert a strong influence on positive psychology research in the coming decades. Some have referred to research at the interface of positive psychology and technology, “positive technology” (Calvo and Peters, 2012; Riva et al., 2012; Villani, 2016; Baños et al., 2017; Gaggioli et al., 2017).

In this review, we do not include technologies related to genetic manipulations or so-called ‘strong’ artificial intelligence (which exhibits general intelligence across domains) because, while, if created, they will be undoubtedly massively influential in well-being research, we believe that their development and effects will be felt most acutely in longer than a decade from the time of this writing (Russell and Norvig, 2016). Similarly, we do not discuss social robotics, the internet of things, or nanotechnology (e.g., Bhushan, 2017) for this reason. On the other hand, we also do not discuss mobile device applications (apps) or wearables (Piwek et al., 2016) because they are already widely used for measurement and intervention, and have been discussed elsewhere (e.g., Kay et al., 2011), nor do we discuss gaming, which has also already been explored for well-being research and interventions (e.g., McGonigal, 2011).

Here, we limit the scope of our focus on emerging technologies that are not yet widespread but which we predict will have a large impact on well-being intervention and measurement within a decade from this writing. In terms of measurement, machine-learning algorithms applied to large data sets (“big data”) are becoming viable, allowing for scalable, unobtrusive measurement of well-being and other psychological constructs at the population level. In terms of interventions, non-invasive brain stimulation, psychopharmacology, and virtual reality are improving rapidly and proliferating in research and consumer contexts, allowing for the modulation of mental states in ways relevant to well-being. This article reviews predictive algorithms, psychopharmacology, non-invasive brain stimulation, and virtual reality, in terms of how each pertain to the near future of well-being research.

## Big Data Machine Learning Predictive Algorithms

“Algorithms… will be so good at making decisions for us that it would be madness not to follow their advice”

– Yuval Noah Harari

Predictive algorithms are perhaps most frequently encountered as continuously improving decision and suggestion systems that decode patterns from millions of user interactions—from Google Now alerting us that a flight is delayed the moment we step out of the door to race to the airport, to the movie suggestions on Netflix. These systems have come into our lives in a manner similar to how GPS did – at first most people tend not to entirely trust the device’s advice, taking its directions more as suggestions. Eventually, though, most people end up trusting and following its directions without much hesitation. Beyond entertainment, marketing, and minor administrative duties, it may soon become the case that predictive algorithms will provide more general insights that resemble those of coaches – and perhaps even make highly accurate predictions about which life decisions will maximize well-being, even in highly personal domains like work and love, both of which have a tremendous influence on well-being.

Algorithms have some learning advantages over human cognition—when an algorithm detects and learns to avoid a mistake based on a few occurrences, an update will push this algorithmic insight to its entire user base. Once a self-driving car learns a new trick, all cars share in the trick—unlike human drivers, who have been trained individually. Algorithm-informed decisions, in short, are on a different cumulative learning curve than human decisions. The quality of their predictions is likely to diverge further and further from those of human cognition with every additional example that is added to its database to digest. In general, algorithms are different from human decision-making in terms of (1) ease of learning, (2) sensitivity and specificity of decision making (typically measured against objective ground-truth), and (3) generalizability of the learning. Humans tend still to have advantages in (3) but machine/algorithmic approaches may soon surpass humans at (1) and (2).

At present, algorithms have not yet entered the highest-stakes domains in the decision-making processes of most people – they are not even close to making career or marriage decisions. However, the data that feeds predictive algorithms in these domains is being collected from most of the population at unprecedented rates, and with all big data algorithms, the strongest predictor of the quality of a prediction system is the availability of data sets of sufficient quality. Mobile phones, owned now by the majority of the global population, are routinely loaded with tens of sensors that span the detection of motion, light, and environmental conditions, and capture health data, like heart rate and step counts. Beyond these sensors, in our use of digital spaces we leave behavioral residues, or “digital footprints,” from sources such as our text message histories, geographic location, and social media posts. With relatively few steps in some cases, these data can be accessed through application programming interfaces (APIs)—well-defined interfaces between algorithms, allowing for the exchange and integration of data into large data ecosystems. In addition, the Quantified Self movement spans many apps and dedicated sensor systems, tracking everything from weight to fluid intake to sleep quality, and pushes the integration of digital quantification forward and into the mainstream (Swan, 2013).

Algorithms can predict various psychological traits of users on the basis of digital footprints with a high degree of accuracy. Kosinski et al. (2013) have shown that “likes” on Facebook can predict sexual orientation, ethnicity, and political orientation – and these algorithm-based personality predictions can exceed in accuracy those made by acquaintances (Youyou et al., 2015). Kosinski and Wang (2017) showed that face-detection algorithms (like those used to unlock smartphones) can distinguish between hetero- and homosexual orientation with 71–81% accuracy, exceeding those of human raters. Beyond these examples using “likes” and images, the vast majority of our digital traces are textual. The linguistic content shared in Facebook posts can be used to predict Big Five personality traits (Park et al., 2015), gender (Park et al., 2016), and the linguistic features used for prediction can reveal interesting features of constructs, such as for religious affiliation (Yaden et al., 2017a). Importantly, a variety of papers have documented the possibility of detecting mental health states from social media (see Guntuku et al., 2017, for a review) as well as physical health issues of communities such as heart disease (Eichstaedt et al., 2015). Across these examples, the predictive power of algorithms often exceeds those of friend ratings or other meaningful baselines currently employed.

**FIGURE 1:**
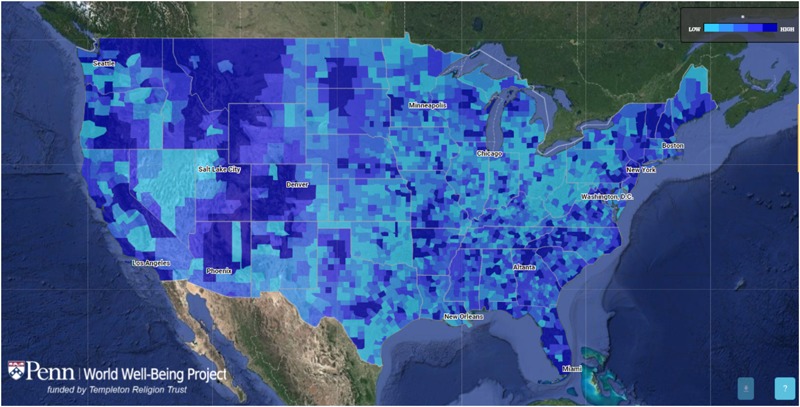
County-level well-being map of U.S. counties generated using a big data analysis of geo-located Tweets (adapted from footnote 1). The low-high dimension represents county level scores of well-being.

So far in psychology, these algorithms primarily constitute an advance in ways to collect data and—using machine learning algorithms-combine distributions of observed features into measurements and estimates. While the accuracy in many of the early studies remains constrained by the self-report scale it is trained on, measurement can proceed at much larger scales unobtrusively. By using machine learning, researchers can train models to associate certain features (i.e., certain words, phrases, or “likes”) with scores on a given psychometric scale or task that as been administered to the same users – but and then the model can predict a score based on the features alone, without the need for traditional measurement (Kern et al., 2016). For example, given sufficient language from Facebook posts from users who have also taken a psychometric self-report scale like a life satisfaction measure, the model can then predict a score on the life satisfaction measure based only on the social media language collected from a sample. In other words, while the training of such a prediction model may require costly survey administration in a sample of users for which social media language is also available, such a model can be applied at much larger scale. For example, a life satisfaction model can be trained on samples of about 2,000 Facebook users, and then applied to the geo-located Tweets of tens millions of Twitter users to generate estimates of average life satisfaction levels across entire geographic regions. As a proof of concept, using this approach, the World Well-Being Project at the University of Pennsylvania has created a map of the well-being of U.S. counties in the United States (see **Figure 1**^1^; see also Schwartz et al., 2013).

In the near future, this increased capability for psychological measurement of individuals and populations will begin to inform and tailor interventions. When algorithms ingesting text messages and social media messages are able to detect, for example, depression relapse or the onset of manic episodes, therapeutic interventions may be initiated immediately. This linguistic approach will likely be combined with other big data streams from genomic and neural network research (e.g., Baker et al., 2014) to increase the accuracy of risk profiles to improve early intervention and may help to guide decisions about which interventions to utilize in a given instance. Health coaching algorithms will be able to give tailored advice on how to increase one’s well-being (e.g., apps might send messages to users like: “sleep more!” “Your correspondence with Alice increases your stress levels!” “Bob calms you down and lifts your mood”). And finally, at the community level, the fine-grained measurement of the psychological health of populations may mean that a number of different policy interventions can be tested in different communities, and the one shown to be most efficacious rolled out on a larger scale—pushing the idea of *evidence-based policy* into the age of big data.

Thus, this technological advance in measurement and algorithm-derived models is progressively identifying possible opportunities for targeted and measurable intervention. We now consider three technologies as candidates to advance the frontier of well-being interventions.

## Psychopharmacology

“… in order to raise global happiness levels we need to manipulate human biochemistry.”

– Yuval Noah Harari

Psychopharmacology is already commonplace in US society and medications for mental disorders are among the most prevalent prescriptions (Kantor et al., 2015). Some research shows that about 70% of Americans are on at least one prescription drug (Zhong et al., 2013) and at least 10% use a psychopharmaceutical (Paulose-Ram et al., 2007). That is to say, psychopharmacology is already quite common in contemporary culture. Because of this proliferation, psychopharmaceutical research is a well-funded and technologically sophisticated domain of modern research. Most of this research has been devoted to treating physical and mental illness, but, increasingly, people are taking medications for less serious mental disorders, which in some cases may come closer to a lifestyle choice than a treatment for a mental disorder. Little is known about the effects of most common pharmaceuticals on well-being, including whether or not there are detrimental side-effects or benefits to well-being.

At some points in US history, taking prescription pharmaceuticals for well-being was popular. Milltown (Meprobamate), for example, was a substance marketed for its capacity to increase happiness. It was found, however, that the substance is addictive and approval for its prescription was withdrawn (Pieters and Snelders, 2009). Psychopharmacology is a well-resourced research field out of which advances in mental health treatment can be expected, and, likely, advances in enhancement. However, the questions of addiction looms large in this area, and it may be the case that, in general, long-term chronic use of many psychopharmaceuticals tends to diminish well-being. Future research in this area will need to play close attention to the difference between addictive “highs” and non-addictive enhancements to overall well-being.

It may also be the case that enhanced well-being is a hitherto understudied mediator of the therapeutic effects of some medications for mental disorders. The most common medications for mental disorders include medications for depression: selective serotonin reuptake inhibitors (SSRIs) and selective serotonin norepinephrine reuptake inhibitors (SSNRIs); anxiety: Benzodiazepines (e.g., Alprazolam and Clonazepam); pain: cyclooxygenase-2 (COX-2) inhibitors and opioids; attention: amphetamine, dextroamphetamine, and methylphenidate. Pain medications have played a central role in an epidemic of opioid addiction (Nelson et al., 2015). Attention deficit medication has been used as an enhancing substance by many college students and has been shown to increase motivation to study though there are health risks to this practice (Smith and Farah, 2011). The question of psychopharmaceuticals for enhancement is a relevant topic in the field of bioethics, and, more specifically, neuroethics (Farah et al., 2004). Again, the relationship between these widely prescribed psychopharmaceuticals and well-being (potential benefits and detriments) is largely unknown.

Chronic versus acute use of psychoactive substances may be an important distinction in terms of well-being outcomes. Several substances currently undergoing clinical trials have demonstrated well-being effects from administrations in single sessions. The first of these is the single session use of Ketamine for the treatment of depression (Murrough et al., 2013), which tends to result in an enhanced sense of well-being (Dillon et al., 2003). MDMA, sometimes referred to as an “empathogen” due to its tendency to increase positive emotions and feelings of warmth toward other people, is currently being tested for its efficacy in treating PTSD (Oehen et al., 2013). MDMA is also reportedly associated with enhanced feelings of well-being (Liechti et al., 2000). Lastly, psilocybin is being tested for its capacity to reduce symptoms of depression and anxiety (Ross et al., 2016). Psilocybin has also been associated with marked increases in aspects of well-being in clinical trials (Griffiths et al., 2006) and correlational studies (Yaden et al., 2016). These substances raise the potential for episodic interventions to treat mental disorders and/or increase well-being. Similar to the self-quantification movement, in the United States there exists a vibrant “biohacking” community that is actively exploring the use of small or below-threshold doses of psychopharmaceuticals to improve daily well-being, attention and creativity (for a discussion, see d’Angelo et al., 2017).

The use of psychopharmaceuticals for enhancement and well-being beyond psychopathology remains relatively uncharted terrain about which further ethical discourse and research is required. This includes research into the potential well-being benefits and detriments of recreational uses of psychoactive substances, which has been rarely explored despite the legality and widespread normative use of various substances (e.g., alcohol, marijuana). With modern efforts to develop “designer drugs” that maximize the effectiveness of psychopharmacology to treat specific problems within specific persons (Belmaker et al., 2012; Oquendo et al., 2014), it is also critical to evaluate under what circumstances these approaches can enhance or diminish well-being.

## Non-Invasive Brain Stimulation

“In research labs experts are already working on more sophisticated ways of manipulating human biochemistry, such as sending electrical stimuli to appropriate spots in the brain…”

– Yuval Noah Harari

Whereas psychopharmacology generally influences many synapses distributed throughout the brain, brain stimulation offers the ability to be more spatially precise. Directly modulating the brain has long been a topic of science fiction, but is now a scientific reality. In *Do Androids Dream of Electric Sheep* (Dick, 1996), Philip K. Dick describes a device called a “Penfield Mood Organ.” This device allows its users to dial up whatever mood or mental state that they desire. It is named for Wilder Penfield, the neuroscientist who demonstrated that running low amplitude electrical current through regions of a patient’s brain during surgery could produce certain reliable effects on one’s subjective experience and bodily functions (Penfield and Boldrey, 1937). By stimulating the motor strip, for example, Penfield could move the patient’s arms or legs; by stimulating temporal lobe he could produce vivid recall of memories. Since the 1980s, brain stimulation technology has been moving Penfield’s pioneering findings closer to the fictional mood-modulating device.

There are several widely used forms of brain stimulation technology, some invasive and some non-invasive. Invasive forms of brain stimulation include deep brain stimulation (DBS; Perlmutter and Mink, 2006) and electroconvulsive therapy (ECT). DBS involves surgically implanting a lead into brain tissue, which can then be activated. DBS has shown breakthrough success in treating Parkinson’s (Moreau et al., 2008) and is being tested as a depression treatment (Mayberg et al., 2005). ECT involves introducing a stronger electrical current that is capable of generating a seizure in patients. It is a widely misunderstood treatment in that many laypeople have a negative impression of it despite its considerable effectiveness in treating major depression (Sackeim, 2017). ECT will remain a standard of care and DBS will likely be shown to demonstrate further efficacy in mental and physical illnesses, but the application of these technologies in well-being research will likely remain limited due to the medical risks involved with these procedures. Closed-loom stimulation may be the most near-term candidate for major advances in invasive brain stimulation (Klein et al., 2016). Furthermore, while Elon Musk’s neuroscience start-up Neuralink may use minimally invasive means in order to create sophisticated brain–computer interfaces (BCI), this possibility may be more than a decade away from immediate relevance to most psychological researchers.

Non-invasive forms of brain stimulation are the kinds of brain stimulation technology that are already beginning to exert an influence in the field of well-being research. Historically, bio- and neurofeedback – the use of operant conditioning in combination with physiological measurement – have shown some efficacy, which is now being enhanced by more precise forms of measurement (Watanabe et al., 2017). Transcranial magnetic stimulation (TMS) involves passing magnetic pulses through the skull and into the cortex, which can then hypo- or hyper-polarize neurons, thereby allowing for some control over the activation or inhibition of particular cortical regions (Hallett, 2000). Transcranial direct current stimulation (tDCS), another form of non-invasive brain stimulation, works by passing a low voltage current between anodal and cathodal electrodes on the scalp, allowing the brain tissue between the electrodes to complete the circuit between them (Fregni and Pascual-Leone, 2007). This electrical charge can raise or lower the action potential threshold in the neurons across particular cortical regions, making them more or less likely to fire, thus altering brain activity across a brain region. TMS and tDCS have both been demonstrated to successfully modulate a number of mental processes, including creativity, morality, learning, attention, and depression (Hamilton et al., 2011).

Most psychological research on TMS has been focused on its application as a depression treatment, for which it has been found effective (O’Reardon et al., 2007), and its application to boosting well-being is only just beginning. Future brain stimulation studies on well-being will build on findings from neuroimaging and optogenetic research. For example, several “hedonic hotspots” have been identified that brain stimulation researchers might target to enhance well-being (Smith et al., 2010). These regions relate to well-being in different ways. For example, “liking” and “wanting” circuits have been differentiated from one another (Kringelbach and Berridge, 2009, 2017).

**FIGURE 2:**
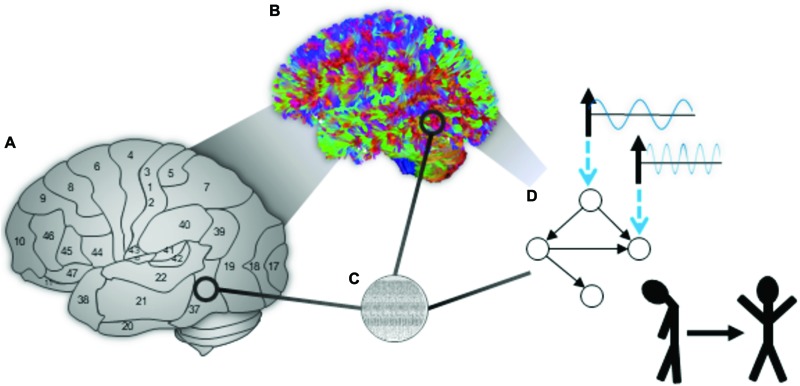
**(A)** The brain includes major neural subdivisions that serve distinct roles in computation, here represented by labeling Brodmann (1909). **(B)** Techniques such as diffusion weighted imaging can provide information about the major connections among brain regions (the “connectome”). **(C)** Low level neural organization supports information processing and is embedded within the macro-scale connectome. **(D)** The brain can be represented by networks at multiple scales that can putatively be guided using control input targeted to specific neurons, regions, and circuits to promote states of better wellbeing. Figure and caption adapted with permission from Medaglia et al. (2017).

In addition to identifying specific targets to influence well-being, brain stimulation is being combined with modern approaches from complex systems to seek personalized approaches to brain stimulation. For example, approaches from modern “connectomics” are identifying ways to control brain dynamics in complex networks (Muldoon et al., 2016). More broadly, control theoretic approaches applied to neuromodulation might lead to energy efficient, adaptable technology that can more precisely guide dynamics to desired neural and cognitive states (see **Figure 2**; Medaglia et al., 2017).

## Virtual Reality

“… it should therefore be theoretically feasible to simulate an entire virtual world that I could not possibly distinguish from the ‘real’ world.”

– Yuval Noah Harari

Virtual reality (VR) involves hardware and software capable of generating realistic sensory simulations (Parsons et al., 2017). While using VR, people experience differing degrees of presence, or the feeling of really being ‘there’ in both mind and body (Riva et al., 2007). During these immersive VR experiences, it is common for virtual stimuli to elicit reflexive responses beyond that which can typically be elicited using 2-D screens and can even evoke responses similar to those produced by equivalent situations in real life (Meehan et al., 2002).

Virtual reality is likely on the verge of becoming a ubiquitous consumer device in economically developed nations. This is primarily due to its capacity to enhance the experience of communication by bringing in a more embodied component, combined with the increasing quality and lowering cost of the necessary hardware. With VR, users can inhabit the same virtual space as their communication partner(s), making the experience more like a face-to-face conversation than current social media platforms allow. The popular social media company Facebook bought one of the most advanced consumer VR companies, Oculus Rift, providing research and development funding to make the widespread use of VR more likely. VR may become a common form of digital interaction, but the effects of spending substantial periods of time in virtual environments for communication or recreation remains unknown, particularly in terms of well-being. Here again, the distinction between addiction, other detrimental effects, and/or benefits to well-being will need to be carefully disambiguated through research.

Positive emotions and other experiences related to well-being can be induced in VR environments. That is, transient VR environments can effectively simulate various specific scenarios, often occasioning visceral subjective responses in users, even in highly controlled laboratory settings. A variety of more general health-related VR applications are currently available (Baños et al., 2013; Freeman et al., 2017) and have been for some time (Riva, 2005; Gregg and Tarrier, 2007). VR is a particularly ideal tool to further create feelings of awe, because most awe stimuli (such as the view from the top of a mountain) are difficult to create in laboratory settings, but are relatively easy to simulate using VR (Chirico et al., 2016). Preliminary studies have indeed found that VR can indeed effectively induce awe in laboratory settings (Chirico et al., 2017). We note that VR is not only a useful well-being research tool for intervention, but also for measurement, as newer equipment will be capable of tracking eye movements and scanning facial expressions. Additionally, physiological measurements can be easily taken in VR laboratory settings and behavior in the virtual environment can be recorded.

Virtual reality technology may have the capacity to democratize certain positive experiences. For example, a hospitalized or otherwise housebound individual could put on a VR headset and walk the streets of Paris, climb Mount Everest, or orbit the planet Earth. Research using VR as a tool for inducing certain emotional states, perceptual illusions, and standardized social interactions will likely become quite common in laboratory contexts. VR can help us understand the specific manipulations that influence well-being with unprecedented control and possibilities. Additionally, the large-scale experiment of VR-mediated communication in social media platforms will become an important topic of well-being research. The well-being of VR users will likely be impacted both by how long they spend in VR and the kinds of experiences they engage in while in virtual contexts.

## Ethical Considerations

“Through trial and error we are learning how to engineer mental states, but we seldom comprehend the full implications of such manipulations.”

– Yuval Noah Harari

The opportunities for well-being measurement and intervention research raised by the emerging technologies reviewed above also raise a number of risks and ethical concerns. These technologies open avenues to an increased degree of control over human experience, both in terms of modifying mental states and traits. This possibility raises concerns about changing personal identity and autonomy as well as issues related to the equitable distribution of technology – issues that are discussed in more detail below. We lack the space to cover the unique issues with each technology, but will make some general observations about issues specifically related to enhancing well-being. As always, safety is paramount, as are protections around informed consent (Iwry et al., 2017). People participants should understand the kind of experience that they are embarking upon in every given instance of enhancement – the risks and the potential benefits. We recommend that research ethics guidelines be developed for each of the technologies reviewed in this article, signed by members of the relevant professional communities (e.g., Madary and Metzinger, 2016).

One perspective on this increased capacity to manipulate human experience has been called ‘Mind Control’ (Medaglia et al., 2017). Neural manipulations combined with modern systems engineering could eventually produce very specific control over mental experience and behavior. While many uses of brain stimulation are in some ways analogous to “nudges” (Thaler and Sunstein, 2008) – altruistic efforts to minimally but effortfully influence individuals to make better choices – it is in principle possible to evoke more potent and specific control over an individual. In the most optimistic cases, we could greatly improve our capacity to enhance well-being in persons. However, the opposite ability to do great harm may also prove possible. Thus, if these techniques become available, we must rigorously evaluate uses of technologies in terms of safety, beneficence, respect for persons, justice, and preservation of human autonomy (Shamoo and Resnik, 2009).

Regardless of the mechanism and potency of an intervention, the possibility of enhancing traits raises the question of which traits might be targeted. A number of traits are highly valued by society, such as intelligence and self-regulation, for the aim of professional and financial success. Some traits have been shown to be highly associated with well-being. The traits related to professional success and well-being form a partially overlapping set. People could be enhanced to have their thresholds for experiencing positive emotions and negative emotions independently adjusted, for example, to alter their overall ratio of emotional experiences (Fredrickson, 2013). These kinds of adjustments, it should be noted, carry the danger of de-coupling one from their assessments of life circumstances. In some cases, one may become more in touch with reality (if they tend to be overly pessimistic) whereas in other cases, there may be a trade-off between realism and well-being, which most people would likely not prefer (see Lavazza, 2016). Specifically, most people desire to have the most accurate possible view of themselves and the world, even at the expense of well-being, as described by Nozick (1974) in his experience machine thought experiment. Therefore, well-being enhancements should also consider and potentially aim to enhance both accuracy and the capacity to pursue one’s goals in the world. Certain personality traits have been associated with well-being, and recent research has shown that personality traits, while usually stable, are changeable (e.g., from a course of CBT; Roberts et al., 2017). Could these interventions change our very identities? If so, how can this possibility be adequately communicated in a robust informed consent process?

In regard to well-being and achievement in particular, one should be concerned with equitable distribution. There are, of course, many resources that are not distributed equitably (e.g., education, healthcare, and the “digital divide” between the haves and have-nots of digital technologies), which is also the case with psychology. ECT, for example, while one of the most effective severe depression treatments, is less available in low SES areas (Sackeim, 2017). More generally, novel technologies developed at great cost are usually more accessible to the affluent. Even if equitable distribution does eventually occur, as it is unlikely to in the early stages of technological development, an enhanced class could emerge that would amplify existing class differences along socio-economic lines. We suggest that researchers and policymakers should not treat technologies for enhancing well-being differently than other resources known to enhance well-being, such as access to educational, vocational, and recreational opportunities. If the approaches to enhancing well-being described above are validated, safe, and become widely available, the principle of equity would dictate that efforts should be made for such interventions to be equitably available to all persons under reasonable safety guidelines.

In addition to trait enhancements, these technologies will increase access to certain kinds of mental states, or experiences. Various intensely altered and meaningful states of consciousness may be amenable to manipulation using these emerging technologies. To illustrate with one kind of mental state that has been shown to be highly associated with well-being, self-transcendent experiences (STEs) are associated with increased connectedness and decreased self-salience (Yaden et al., 2017b). A number of mental states described in common psychological constructs contain a self-transcendent aspect (though they are otherwise more dissimilar than alike), including: flow (Csikszentmihalyi, 1996), mindfulness (Davidson and Kabat-Zinn, 2004), awe (Keltner and Haidt, 2003), and mystical experiences (Hood et al., 2001). There is variability in our culture in terms of who has had these experiences and who has the time and resources to seek them out. Several studies have shown that about 30% of the population completely agrees that they have felt at one with all things (Hood et al., 2009). Some of these experiences are counted among life’s most meaningful moments and psychopharmacology and non-invasive brain stimulation may make these experiences increasingly available. This possibility raises a host of ethical concerns, especially in cases where direct technological causation is incompatible with one’s metaphysical commitments and given the possibility that easy access to such experiences might diminish their value or positive effects. Such mental states, which impact well-being as well as identity and interact strongly with belief and value systems, will likely be amenable to manipulation by emerging technologies, so ethical discussion regarding this possibility is warranted and needed. Describing a vision that seems equal parts inspirational and worrying, Harari writes:

In the future, however, powerful drugs, genetic engineering, electronic helmets and direct brain-computer interfaces may open passages to these places. Just as Columbus and Magellan sailed beyond the horizon to explore new islands and unknown continents, so we may one day embark for the antipodes of the mind.

In the case of measurement issues and the increasing power of algorithms, it should be acknowledged that business ecosystems exist to generate economic value from these datasets. Many companies already collect, integrate, and trade such data as a core part of their business model, and micro-targeted advertisement on the basis of such data sets was at work in the last few US elections (Grassegger and Krogerus, 2017) with unclear effect. The need for ethical discourse and education about these existing technologically driven changes to social processes is urgently necessary. Furthermore, well-being research more generally could be leveraged by business ecosystems: if well-being is an in-demand product of some technologies, these technologies are likely to become commercialized and subject to general market principles. This is already the case for numerous non-invasive brain stimulation technologies well in advance of scientific consensus about the efficacy (or lack thereof) of these products. While there may not currently be a regulatory gap for these early technologies (Wexler, 2016), new developments should be closely monitored to ensure that business practices conform to federal guidelines, and consumers should be educated about the evidential basis for specific technologies. Researchers as well as the public ought to be vigilant against false claims in the media or even in popular fiction (Wurzman et al., 2017) regarding the efficacy of well-being enhancement. Consideration of the concerns and risks raised in this section such as consent, safety, beneficence, respect for persons, justice, autonomy, and equitable distribution, as well as other ethical considerations that we cannot yet anticipate is imperative. We also note, however, that failing to take the potential utilities of such technologies into account raises a set of ethical concerns (e.g., Danaher et al., 2018).

## Conclusion

New technologies capable of enhancing measurement and intervention will likely have a sizable impact on the science of well-being. To reiterate, we are not advocating for the increased utilization of technology for the purpose of enhancing well-being, nor are we implying the naïve view that a positive correlation between technology use and well-being exists. In fact, it may well be the case that well-being research finds that in many cases less frequent use of certain technologies increases well-being, as has been found in the area of some kinds of social media use, for example (Seabrook et al., 2016). A careful empirical evaluation of each intervention is warranted in terms of its capacity to increase or decrease well-being – an effort that requires larger and richer datasets than have been available to field. The extent to which biotechnology and information technologies achieve safety, effectiveness, and, especially, ethical aims, must form the core criteria for the introduction of any technology into our lives for the purpose of increasing well-being.

## Author Contributions

DY conceived of and wrote the manuscript. JM and JE contributed writing, figures, and edits.

## Conflict of Interest Statement

The authors declare that the research was conducted in the absence of any commercial or financial relationships that could be construed as a potential conflict of interest.

## Footnotes

1 map.wwbp.org
